# Supplementary material for: Dynamic machine vision with retinomorphic photomemristor-reservoir computing
Source: Nat Commun. 2023 Apr 15;14:2169. doi: 10.1038/s41467-023-37886-y (PMC10105772; doi:10.1038/s41467-023-37886-y)
Supplement: Supplementary file 1 — Supplementary Information [file 41467_2023_37886_MOESM1_ESM.pdf]

Supplementary Information

Dynamic machine vision with retinomorphic photomemristor-reservoir computing

Hongwei Tan* and Sebastiaan van Dijken*

* e-mail: hongwei.tan@aalto.fi; sebastiaan.van.dijken@aalto.fi

Supplementary Note 1. Details of the retinomorphic photomemristor-reservoir computing (RP-RC) system

The ITO/ZnO/NSTO photomemristors are reconfigurable because of controllable (photo)electron trapping and de-trapping by defect states (oxygen vacancies) at the ZnO/NSTO interface. They can operate as a diode, photovoltaic cell, photodetector, memristor, or photomemristor, depending on the required task. The output *y* mainly depends on the applied bias voltage (enabling sensory gating), the present state *h*, and the optical input *X* (Fig. 1a):

$y_t = f\,(V, h_t, X_t)$

In this work, we used the ITO/ZnO/NSTO junctions as photomemristors, combining conventional photodetection and memristive behavior with built-in memory. To demonstrate the unique advantage of photomemristive properties for dynamic vision processing, we compared conventional visual sensing and photomemristive visual sensing using the same 5 × 5 PMA. In conventional sensing mode, the PMA uses the peak values of the photoresponse (Supplementary Fig. 1b) for further processing as conventional image sensors do. In photomemristive sensing mode, memristive states of the photoresponse (Supplementary Fig. 2) are used for further processing. Supplementary Fig. 9 shows results for the detection of videos playing the words 'APPLE', 'LIME', 'OLIVE', 'DATE', and 'GRAPE' using conventional visual sensing without hidden states (upper panels) and photomemristive sensing with hidden states (lower panels). In the latter configuration, the ITO/ZnO/NSTO junctions operate as retinomorphic sensors. Next, we used these two data sets to train identical classification networks with a Gaussian noise factor of 0.30. A high classification accuracy of 91.3% is obtained when the PMA works as retinomorphic sensor with hidden states (Supplementary Fig. 10c and 10d). In comparison, the accuracy is only 36.2% when the PMA operates as a conventional visual sensor without hidden states (Supplementary Fig. 10e and 10f).

Supplementary Note 2. Calculation of visual decision maps based on MRP

Car visual decision:

if $x_{robot} < x'_{robot} < x_{robot} + W$:

slow down

else:

keep speed

Robot visual decision:

if $x_{car} < x'_{car} < x_{car} + L$:

slow down

else:

keep speed

Here, the x'_{robot} and the x'_{car} indicate the predicted positions of the robot (at $t = x_{car}/v_{car}$) and car (at $t = x_{robot}/v_{robot}$) and can be described as:

$$x'_{robot} = v_{robot} \times x_{car}/v_{car}$$

$$x'_{car} = v_{car} \times x_{robot}/v_{robot}$$

As a result of the above calculation, the visual decision maps of the car and the robot are shown in Fig. 4e and 4f.

Supplementary Figures

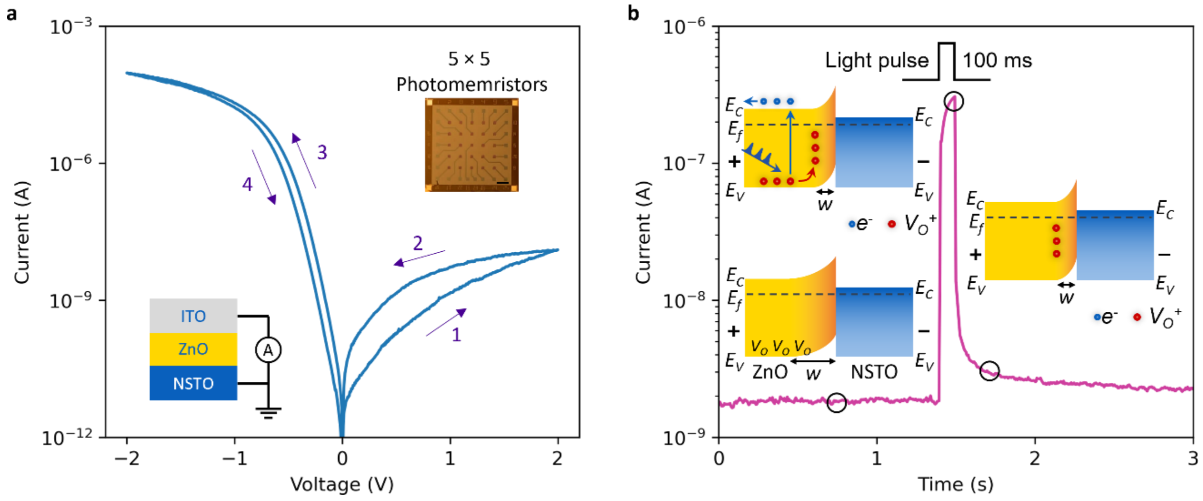

Supplementary Figure 1. Characterization of the photomemristor. a Electrical resistive switching behavior of the ITO/ZnO/NSTO photomemristor structure. The insert shows an optical image of a 5 × 5 photomemristor array (scale bar indicates 500 μm). During the measurement, a voltage is applied to the ITO film with the NSTO substrate grounded. **b** Excitatory postsynaptic current (EPSC) response of the photomemristor to a blue light pulse with a duration of 100 ms and an amplitude of 0.65 mW mm^{-2}. The high on/off ratio ($> 10^2$) enables access to a broad range of rich dynamic analog states. The photomemristive response originates in optically and electrically controlled charging and migration of oxygen vacancies near the ZnO/NSTO interface[1]. The insert shows a schematic of the photomemristive mechanism. Illumination of the photomemristor reduces the Schottky barrier width at a positive bias voltage (1 V on ITO) as the positively charged oxygen vacancies (V_O^+) generated by the incident photons migrate to the interface area. This photo-detrapping process narrows the barrier and increases the electrical conductance. When light is turned off, photoelectrons are no longer generated, but the V_O^+ remains at the interface for some time, resulting in persistent dynamic memory states.

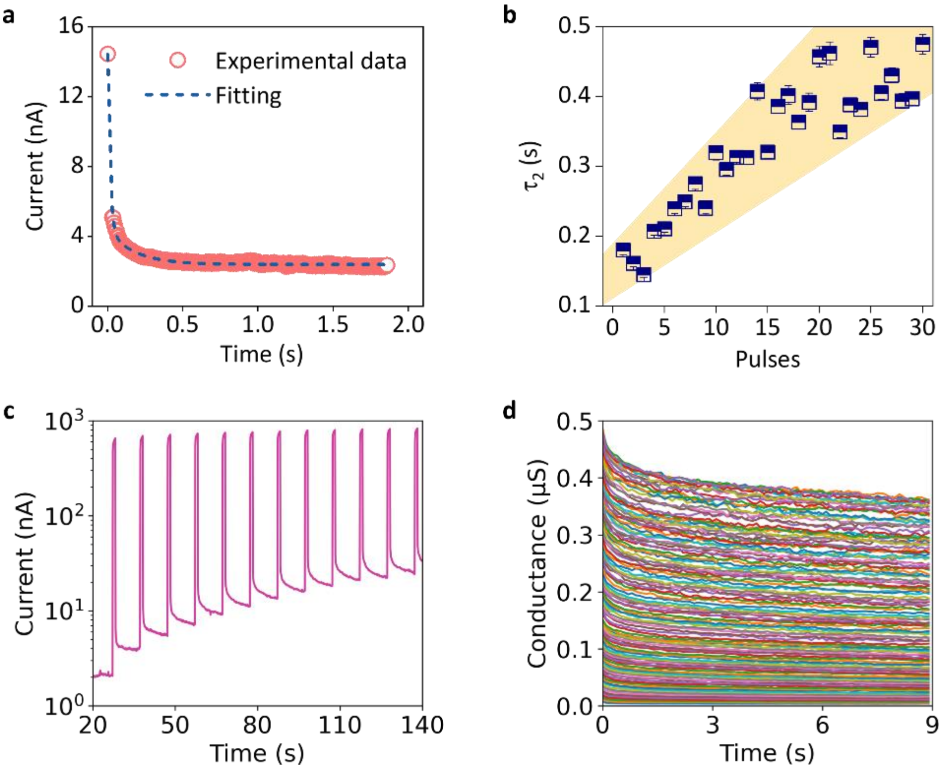

Supplementary Figure 2. Dynamic decay of the photomemristor current and analog states of the ITO/ZnO/NSTO structure. a Exponential decay of the photomemristor current after a single 1 s optical pulse. The dashed line is a fit to the experimental data using an exponential decay function. **b** Dependence of the decay constant τ on the number of optical pulses (pulse duration 1 s, repetition rate 0.1 Hz), demonstrating pulse number dependent dynamics, which is required for dynamic vision reservoirs. The error bars represent standard deviations. **c** Evolution of the photomemristor current during an optical pulse sequence (pulse duration 1 s, repetition rate 0.1 Hz). **d** Dynamic conductance states (126) of the PMA measured after applying 1 to 126 optical pulses. The parameters are the same as in **c**.

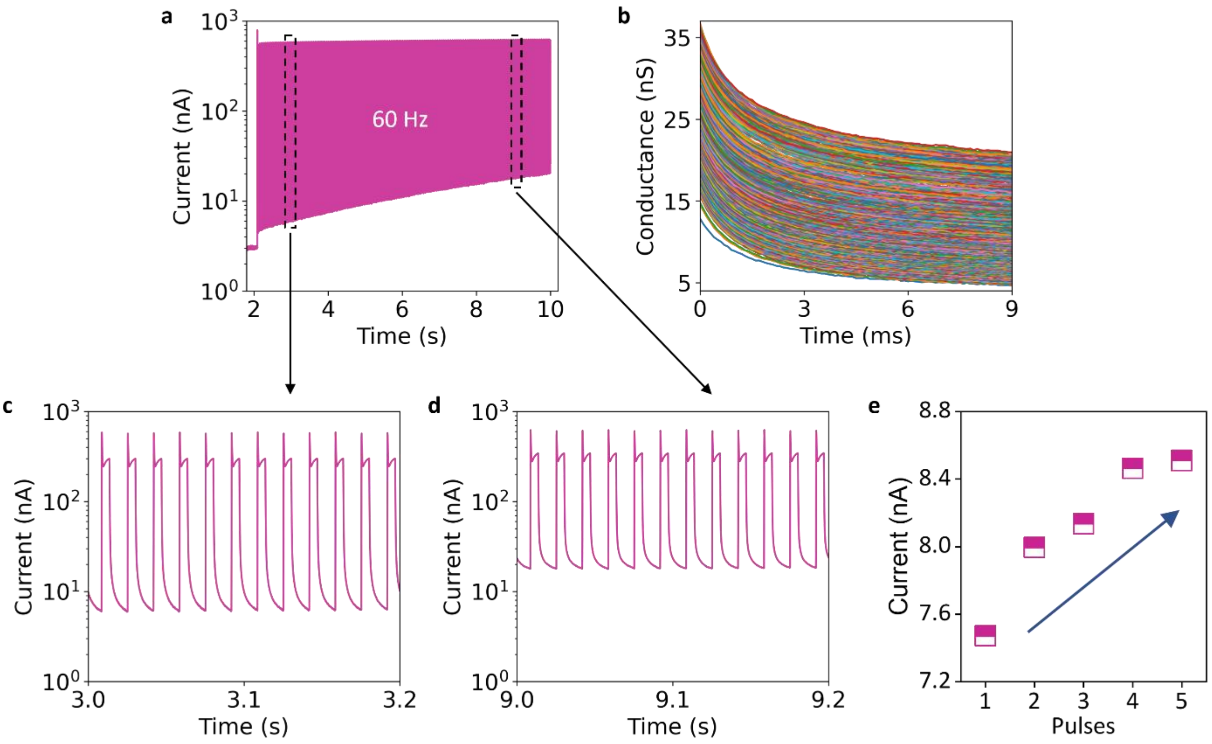

Supplementary Figure 3. Photomemristive switching behavior at 60 Hz, corresponding to 60 frames per second (fps). a Sensing, accumulating, and memory of optical inputs (*X*) using 5 ms pulses at a repetition rate of 60 Hz and a photomemristor bias voltage of 1.0 V. The photomemristor senses and temporally memorizes optical information through the slowly decaying photomemristor current. **b** More than 400 dynamic analog hidden states of the photomemristor measured after applying repeated optical pulses with a duration of 5 ms and at a repetition rate of 60 Hz. **c**, **d** Details of the photomemristor response recorded at 3 s and 9 s in **a**. **e** Current memory states for the first five optical pulses, indicating distinguishable and increasing memory with pulse number. The values are extracted from the measurements by averaging the current over 5 ms after each pulse.

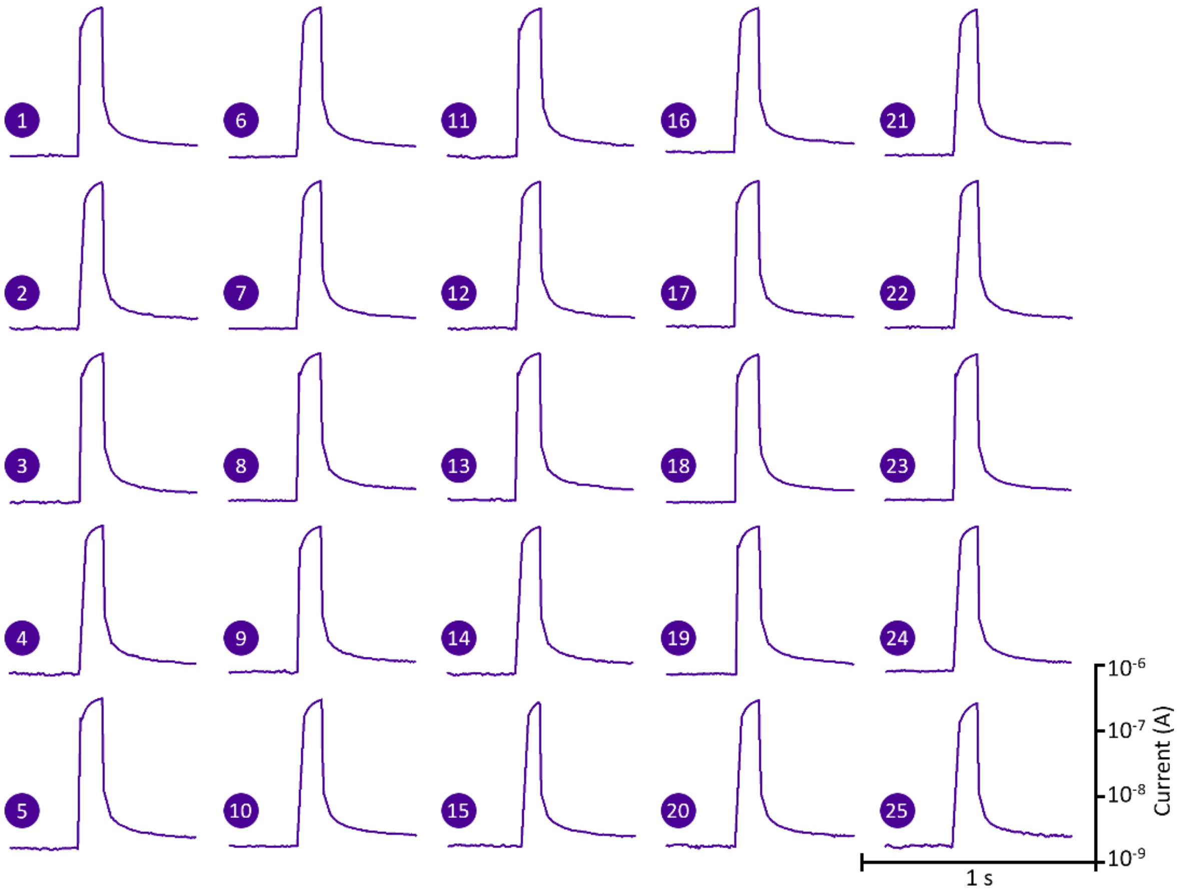

Supplementary Figure 4. Typical excitatory postsynaptic current response of the 5 × 5 photomemristor array (PMA). The duration of the light pulses is 100 ms.

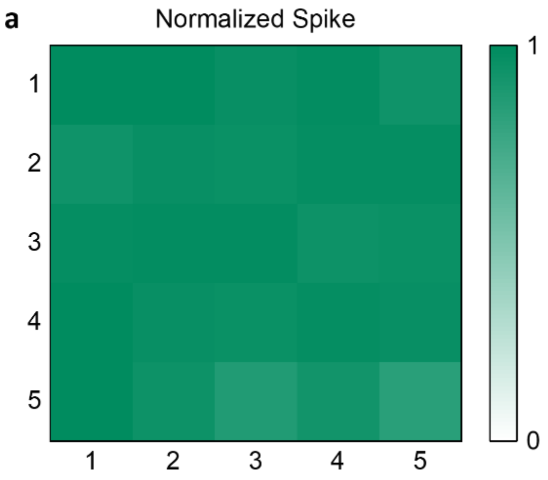

Supplementary Figure 5. Uniformity of the photocurrent response in the 5 × 5 photomemristor array (PMA). a Normalized spike values of the peaks in the excitatory postsynaptic current response of the PMA shown in Supplementary Fig. 4. **b** Normalized current states measured 0.1 s after illumination by a 100 ms light pulse. Both uniformity maps are extracted from Supplementary Fig. 4.

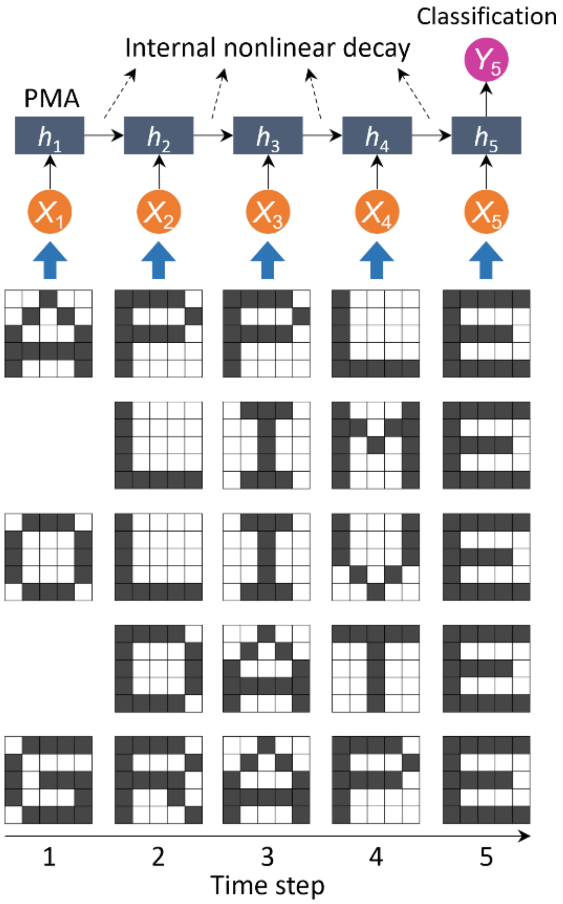

Supplementary Figure 6. Schematic illustrating recognition of videos that play words letter-by-letter. Videos playing the words 'APPLE', 'LIME', 'OLIVE', 'DATA', and 'GRAPE' letter-by-letter (100 ms duration per frame at 2 Hz rate), which all end with the letter 'E', are used as video input to the retinomorphic PMA. The output currents of the PMA decay exponentially. Only the photomemristor currents of the last frame (h_5) recorded after playing the letter 'E' are used as features for recognition by the readout network.

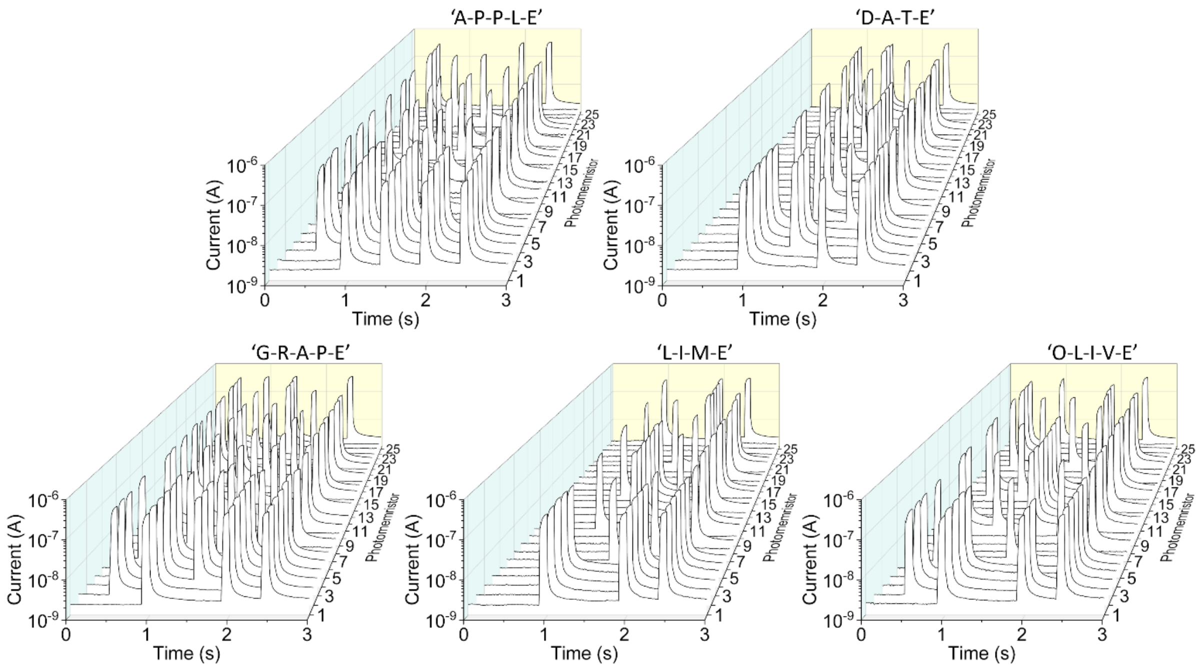

Supplementary Figure 7. Output currents of the PMA when playing ‘APPLE’, ‘LIME’, ‘OLIVE’, ‘DATA’, and ‘GRAPE’ letter-by-letter. The duration of the optical pulses is 100 ms.

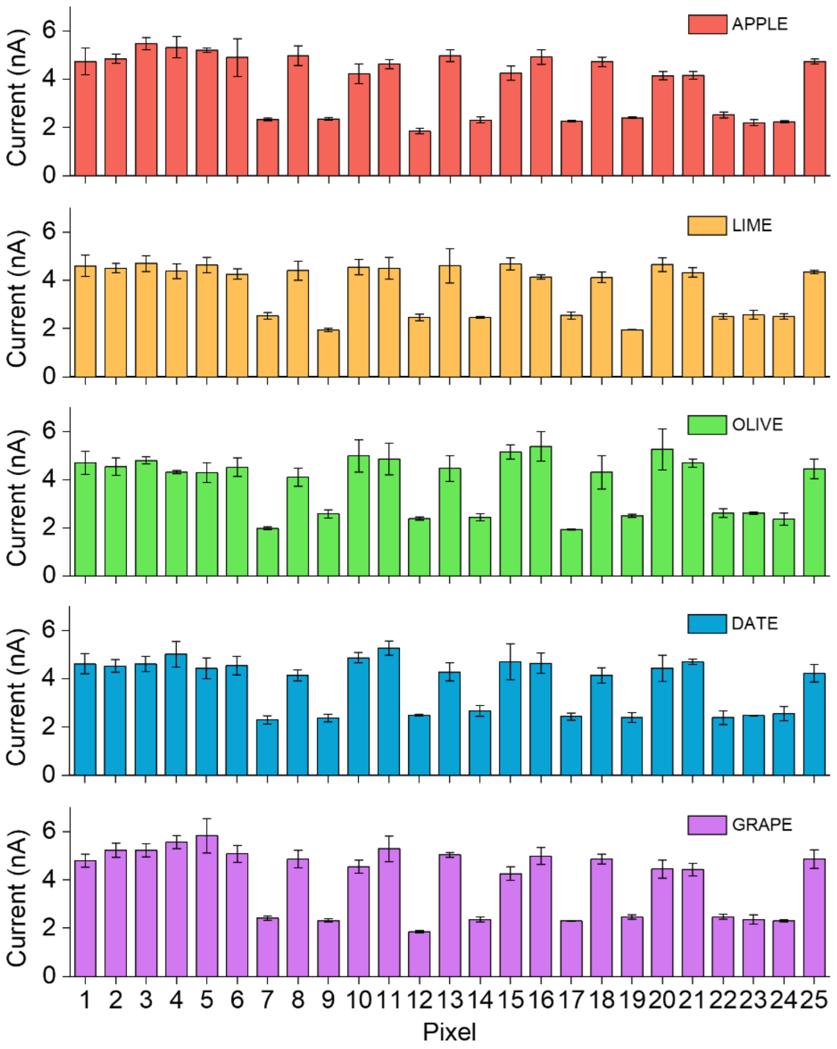

Supplementary Figure 8. Output currents of the 25 photomemristors in the 5 × 5 PMA after playing the last letter 'E' of the words 'APPLE', 'LIME', 'OLIVE', 'DATA', and 'GRAPE'. The photomemristor currents contain information about the last letter 'E' and hidden spatiotemporal information about all previously played letters. The output currents of the PMA are therefore different for the five words. The hidden states are recognized as words by a readout network in the RP-RC system. The error bars indicate the standard deviation of three repeated measurements.

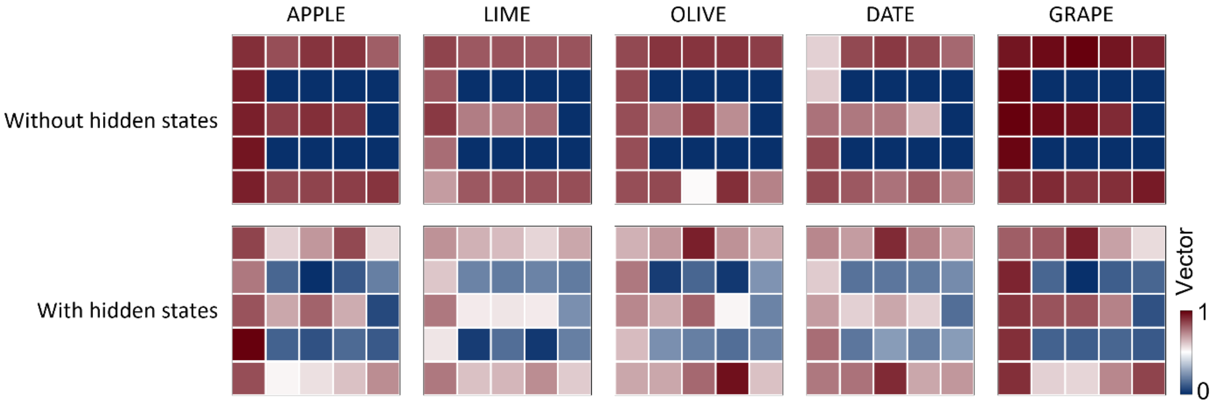

Supplementary Figure 9. Output feature vectors of the 5 × 5 PMA after detecting videos playing ‘APPLE’, ‘LIME’, ‘OLIVE’, ‘DATE’, and ‘GRAPE’ letter-by-letter. The upper panels indicate the output of the PMA if it operates as conventional image sensor (reading peak values of the photoresponse) without hidden dynamic states. The lower panels indicate the output of the PMA if it works as retinomorphic sensor (reading memristive states) with hidden dynamic memory. Although all output images show the last letter ‘E’ of the five videos, the images in the lower panels are different from each other because they contain hidden spatiotemporal information of the previously played letters. This built-in photomemristive effect facilitates accurate video recognition (results in Supplementary Fig. 10).

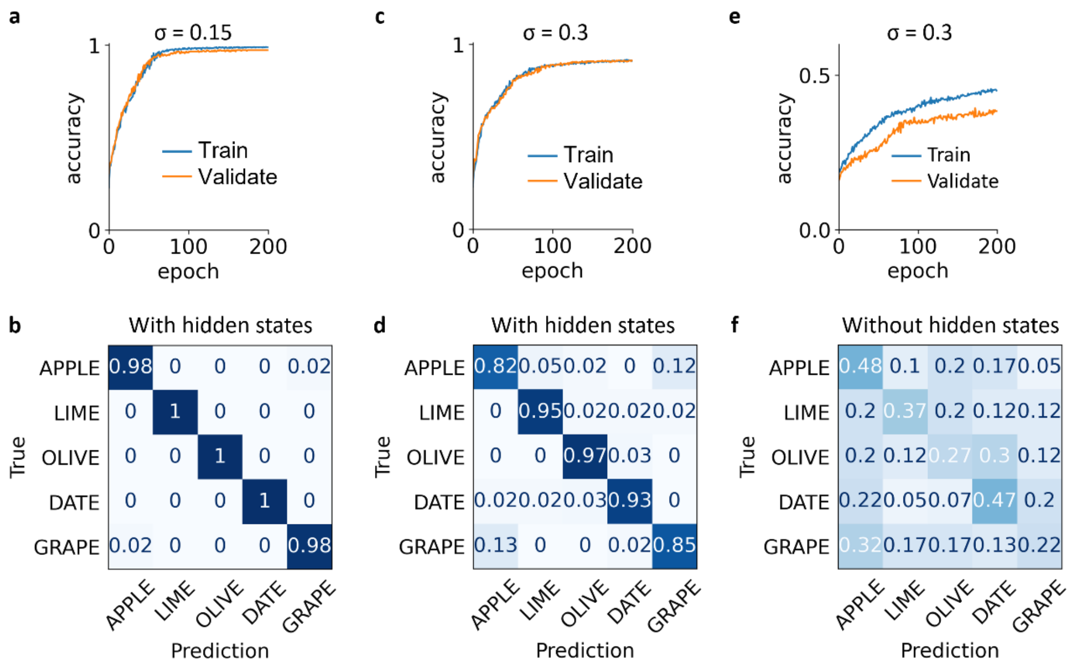

Supplementary Figure 10. Comparison of accuracies with different Gaussian noise (σ) and with/without hidden memory states. a, **b** Training and validation accuracy (~97.3%) of the readout network and confusion matrix for video recognition tasks with Gaussian noise factor σ = 0.15. The 2% misrecognition of 'APPLE' and 'GRAPE' is explained by both words containing the letters 'A', 'P', and 'E'. **c**, **d** Training and validation accuracy (~91.3%) of the readout network and confusion matrix for the same video recognition tasks with Gaussian noise factor σ = 0.30. The ~15% misrecognition of 'APPLE' and 'GRAPE' is again explained by both words containing the letters 'A', 'P', and 'E'. In (a) – (d), the PMA works as a retinomorphic sensor, wherein dynamic memory states are used for video recognition. **e** Training and validation accuracy (~36.2%) of the same readout network when the PMA works as conventional image sensor (using peak values of the photoresponse shown in Supplementary Fig. 1b) without hidden states (upper panels of Supplementary Fig. 9). The accuracy values are much lower than that in **c**. **f** Corresponding confusion matrix of test video recognition with conventional photodetection, showing lower validation accuracy (22% – 48%) compared to **d**. The results in this figure demonstrate the importance of dynamic hidden states for dynamic vision perception.

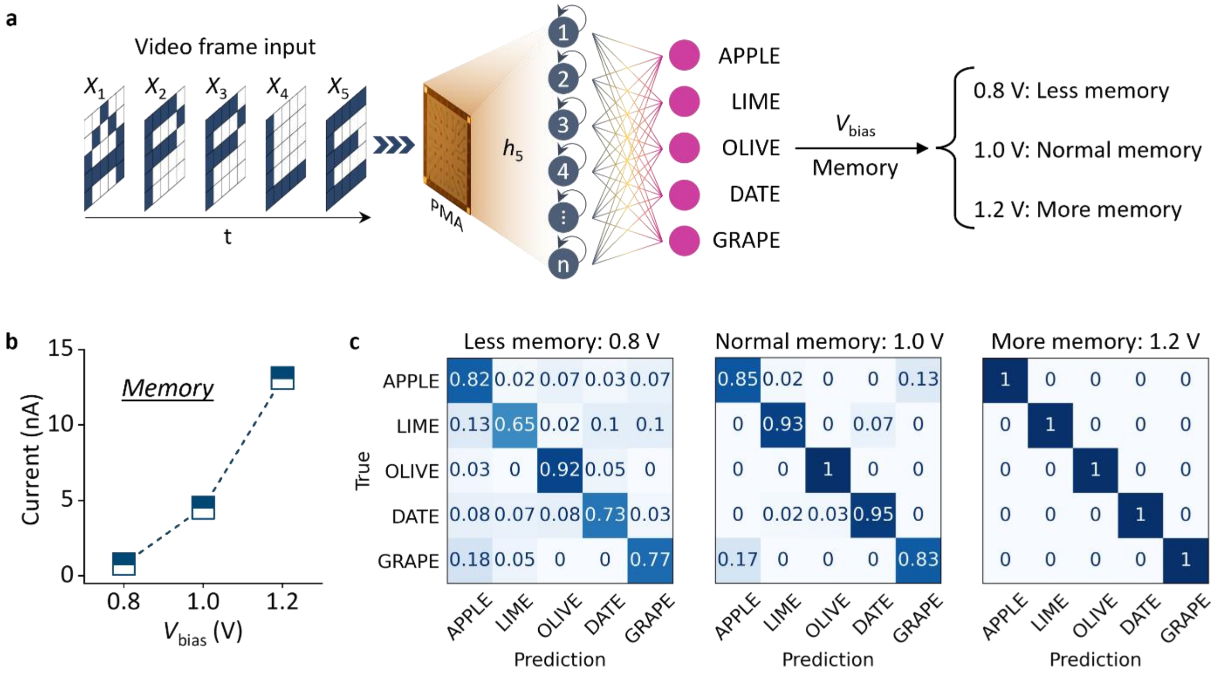

Supplementary Figure 11. Memory-dependent video recognition accuracy. a Schematic diagram of the RP-RC system with bias voltage dependent memory for recognition. The bias voltages (V_{bias}) are 0.8 V, 1 V, and 1.2 V, corresponding to low, medium, and high memory. **b** Photomemristor output currents (memory states) recorded at different bias voltages. The light pulses illuminate the PMA for 100 ms and the frame-to-frame rate is 2 Hz. The current states are read after the 5th pulse shown in Fig. 2f. The memory states increase with increasing bias voltage. **c** Confusion matrix of video recognition demonstrating an increase in test accuracy from 78% to 100% when the bias voltage is enhanced from 0.8 V (low memory) to 1.2 V (high memory).

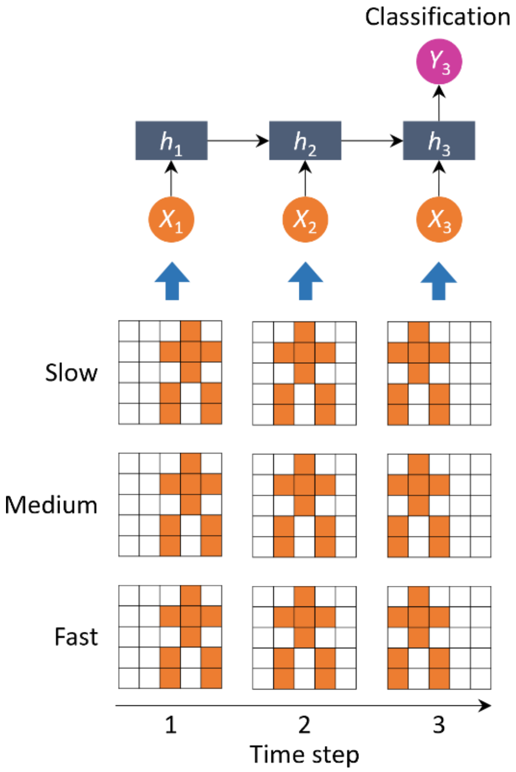

Supplementary Figure 12. Schematic illustrating speed recognition. Programmed optical inputs playing the motion of an object at different speeds (slow (3 s), medium (1.5 s), fast (0.6 s) for all 3 frames). The duration of each frame is 50 ms. The three motions are used as optical input to the retinomorphic PMA. Only the photomemristor currents recorded after playing the last frame of motion (h_3) are used as features for recognition by the readout network.

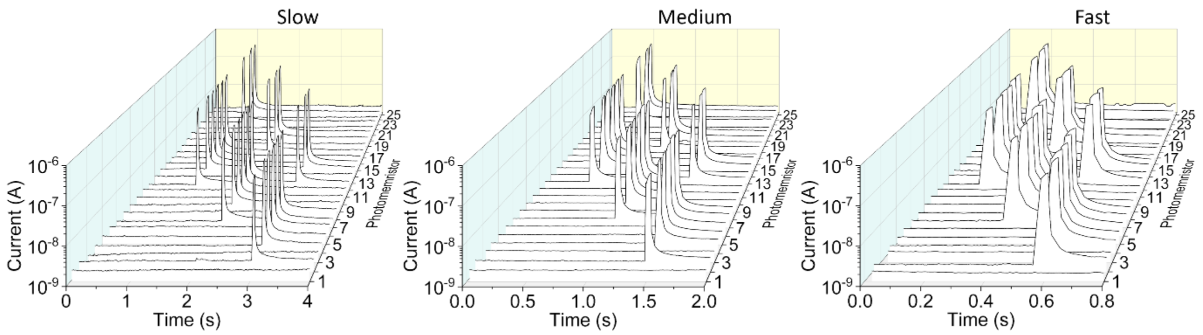

Supplementary Figure 13. Output currents of the PMA when detecting a motion at three different speeds. The duration of the programmed optical signals is 50 ms.

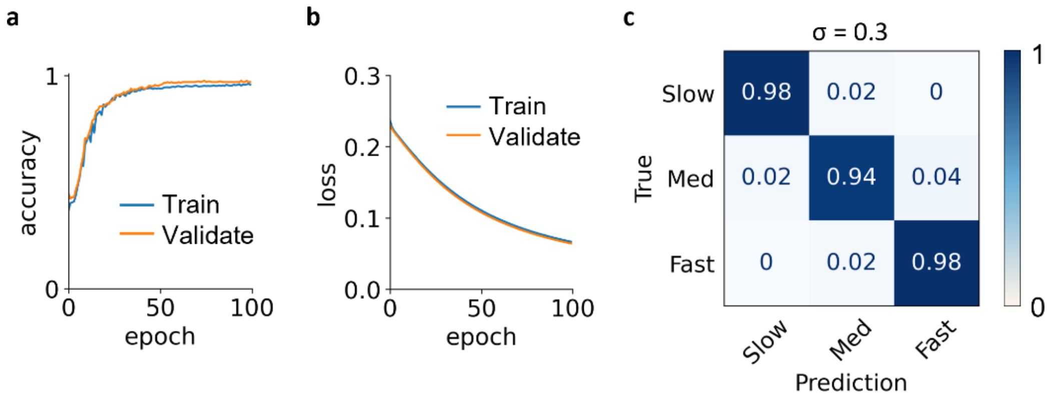

Supplementary Figure 14. Results of motion recognition with a noise factor of 0.30. An accuracy of 97% **a** and loss < 0.1 **b** are obtained when the readout network is trained with data noise factor $\sigma = 0.30$, indicating accurate recognition of motion speed. **c** Corresponding confusion matrix of motion recognition.

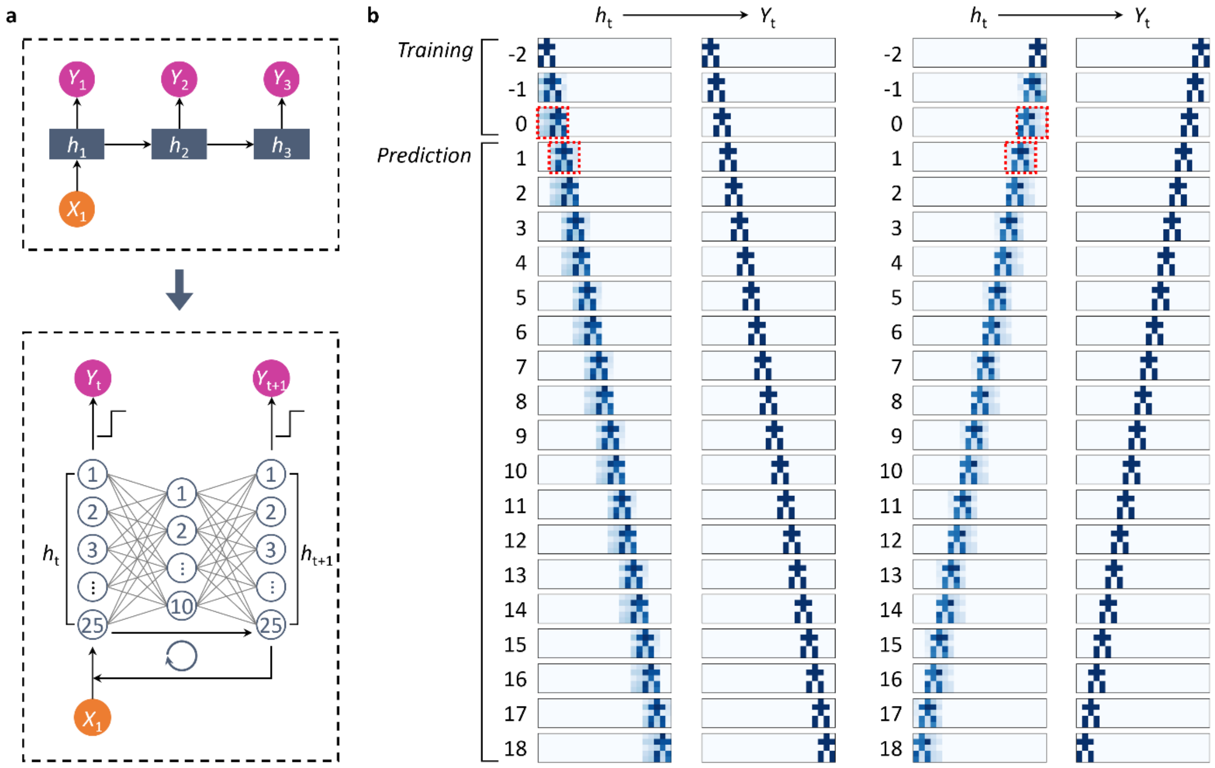

Supplementary Figure 15. Autoencoder and prediction results. a Schematic of the prediction network with autoencoder. The autoencoder has 25 inputs corresponding to the 25 PMA pixels, 10 hidden representations associating input and prediction, and 25 outputs corresponding to the predicted future frames (h_{t+1}) with the same dimension as the input frame (h_t). After training, upon receiving the first frame of motion, the next frames are predicted by repeated feedback input. **b** Predicted output motion images h_t with hidden memory are transformed into Y_t by applying a simple step function (f(x) = 0 (if x < 0.5) or 1 (if x ≥ 0.5)) on h_t. To extend the field of view, we introduced a shifting operation (red dash box in time step 0 and 1) to simulate eyeball movement or head rotation towards the target[2]. This shifting operation enables the continuous prediction of motion.

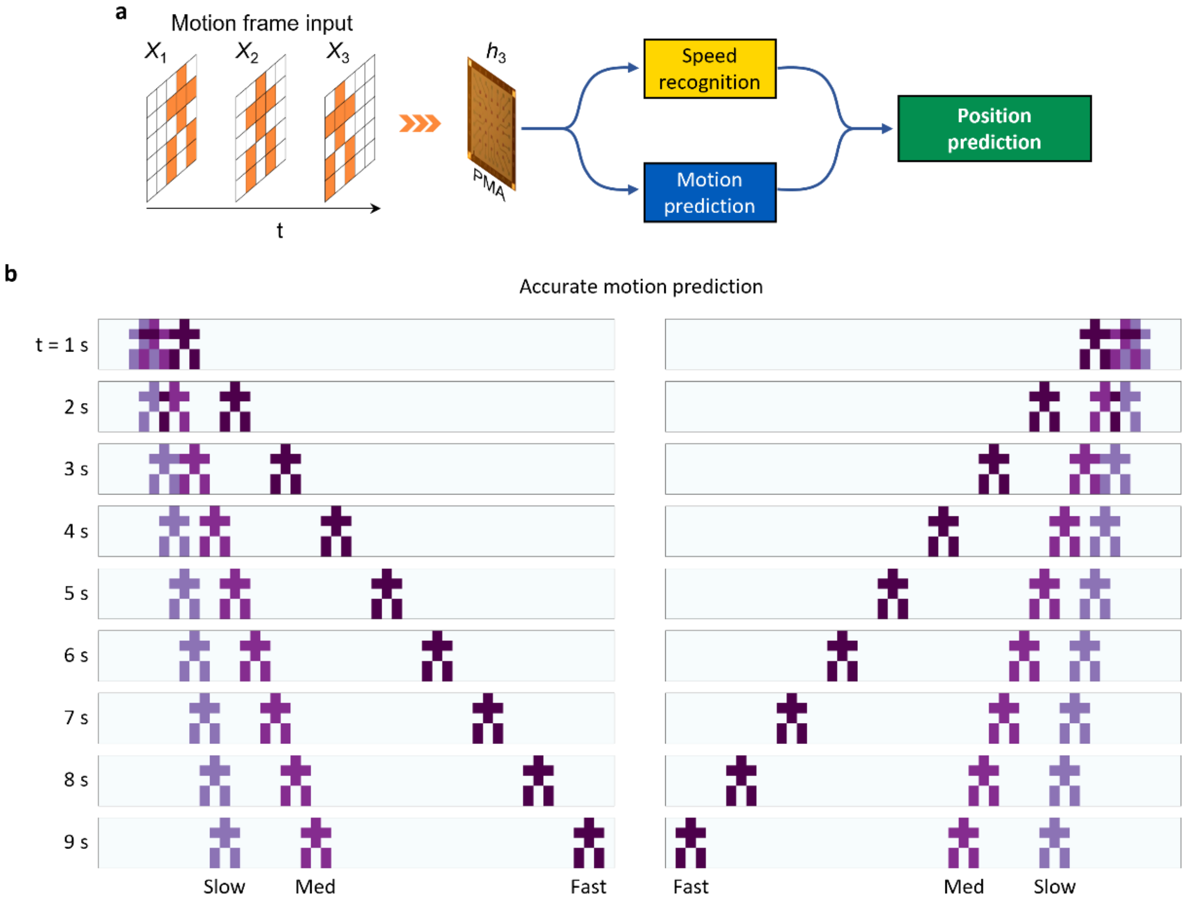

Supplementary Figure 16. Position prediction by combining speed classification and motion prediction. a Schematic diagram of the position prediction process. Speed recognition is used to calculate the total number of motions steps during a certain time and motion prediction projects the trajectory of the object. By combining the results of the two networks, the position of the object is predicted accurately at any time. **b** Prediction results for a person moving to the right/left. The first 9 s are show as an example.

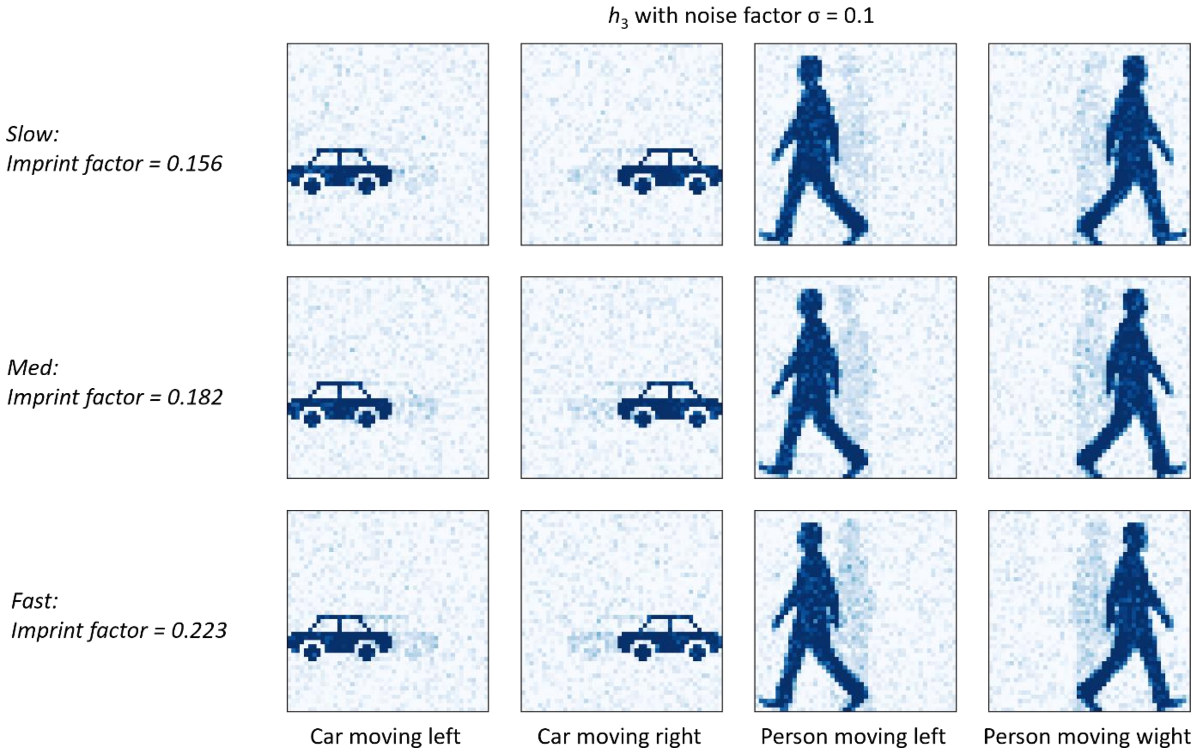

Supplementary Figure 17. Generated training dataset for speed classification of a robot and a car. The merit of dynamic hidden memory in the PMA was used as basic rule to generate the dataset. The parameters of imprint factors of previous frames were added to common images. The imprint factors are 0.156 for slow motion speed, 0.182 for medium motion speed, and 0.223 for fast motion speed. All the values are extracted from the experimental data shown in Fig. 3c. Additionally, 10% noise was used for the generation of the dataset.

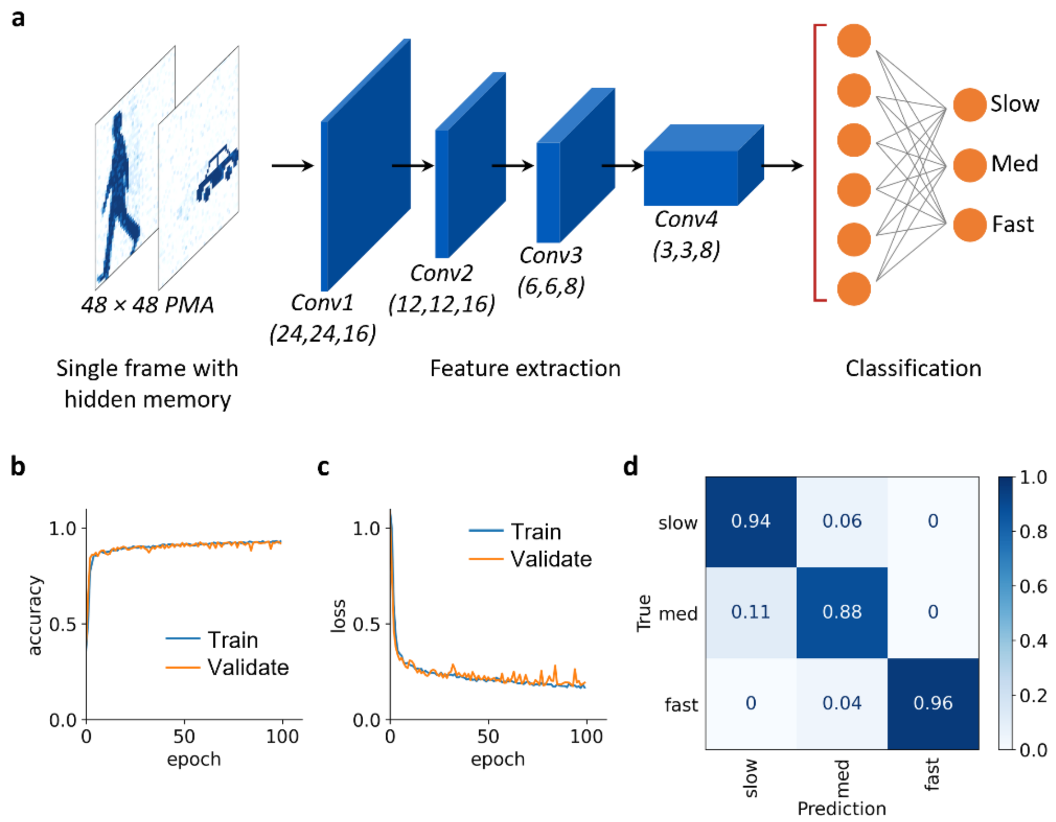

Supplementary Figure 18. Classification of the motion speed of a robot and a car with different moving directions using a convolutional neural network (CNN). a Structure of the CNN, which has 4 Conv2D layers and 4 MaxPooling2D layers to extract the features in the present frame (h_3) with hidden memory states of the previous frames. A fully connected layer was used to classify the features. High training and validation accuracy **b** and low loss **c** are obtained, indicating accurate classification of the motion speeds irrespective of the direction of motion. These results are only possible because of the inherent dynamic memory of the PMA. Without hidden memory states, it would be impossible to predict the speed using the last informative frame. **d** Confusion matrix of the test accuracy.

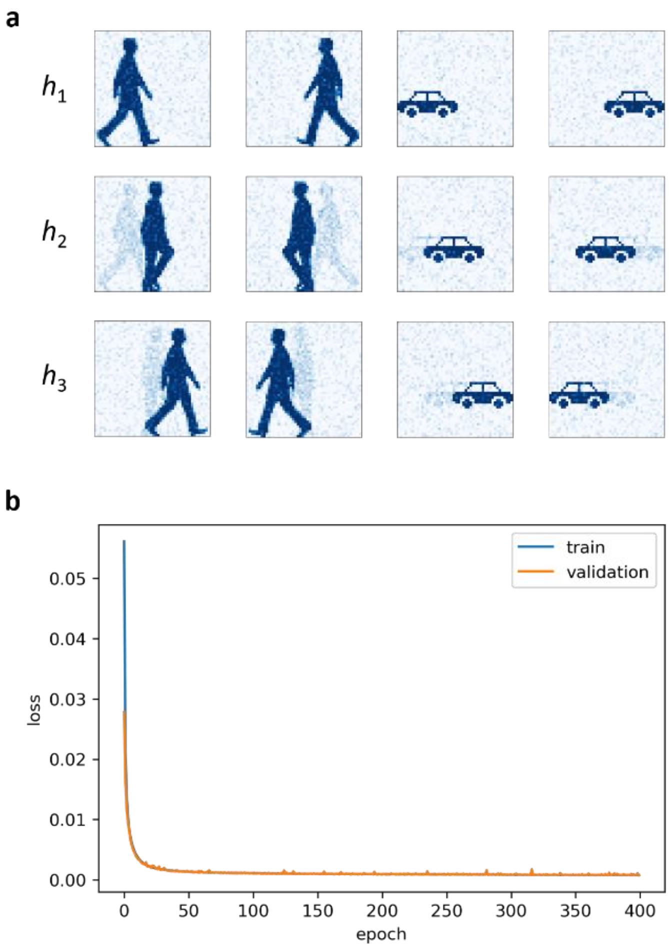

Supplementary Figure 19. a Typical training dataset. The images are generated by adding 10% Gaussian noise to the first three frames (h_1, h_2, h_3) of each motion. The motions include a person or a car moving right or left. Hidden memory of previous positions is included. For example, the h_2 frame contains imprint of motion h_1, and h_3 contains imprints of motion h_2. The generated datasets were used to train the CAE (Fig. 4b) During training, h_1 and h_2 were input under the supervision of h_2 and h_3. More details about training can be found in Methods. **b** Training loss of the convolutional autoencoder (CAE) shown in Fig. 4b.

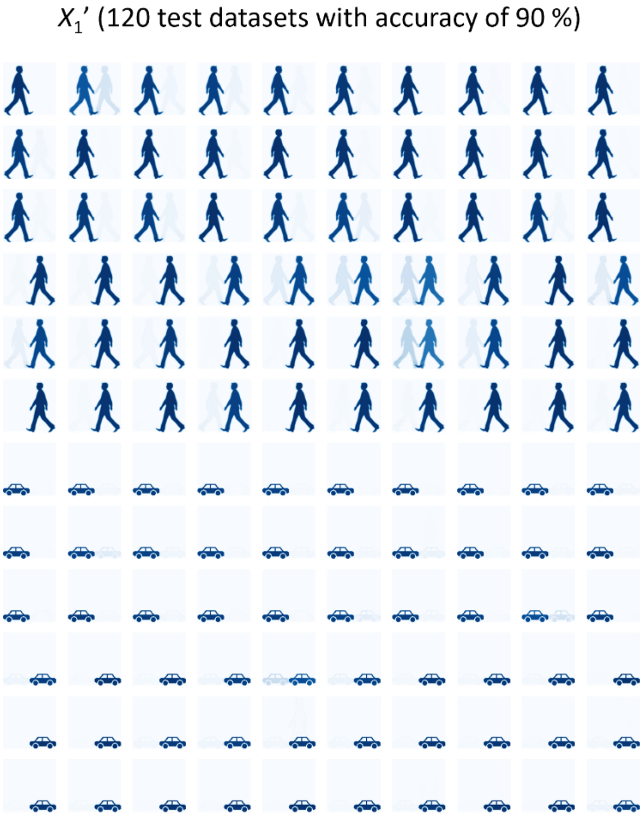

Supplementary Figure 20. Audio-to-first frame (X_1') crossmodal recognition. Predicted images (X_1') for 120 audio inputs to the DNN. The test accuracy is 90% for a random dataset.

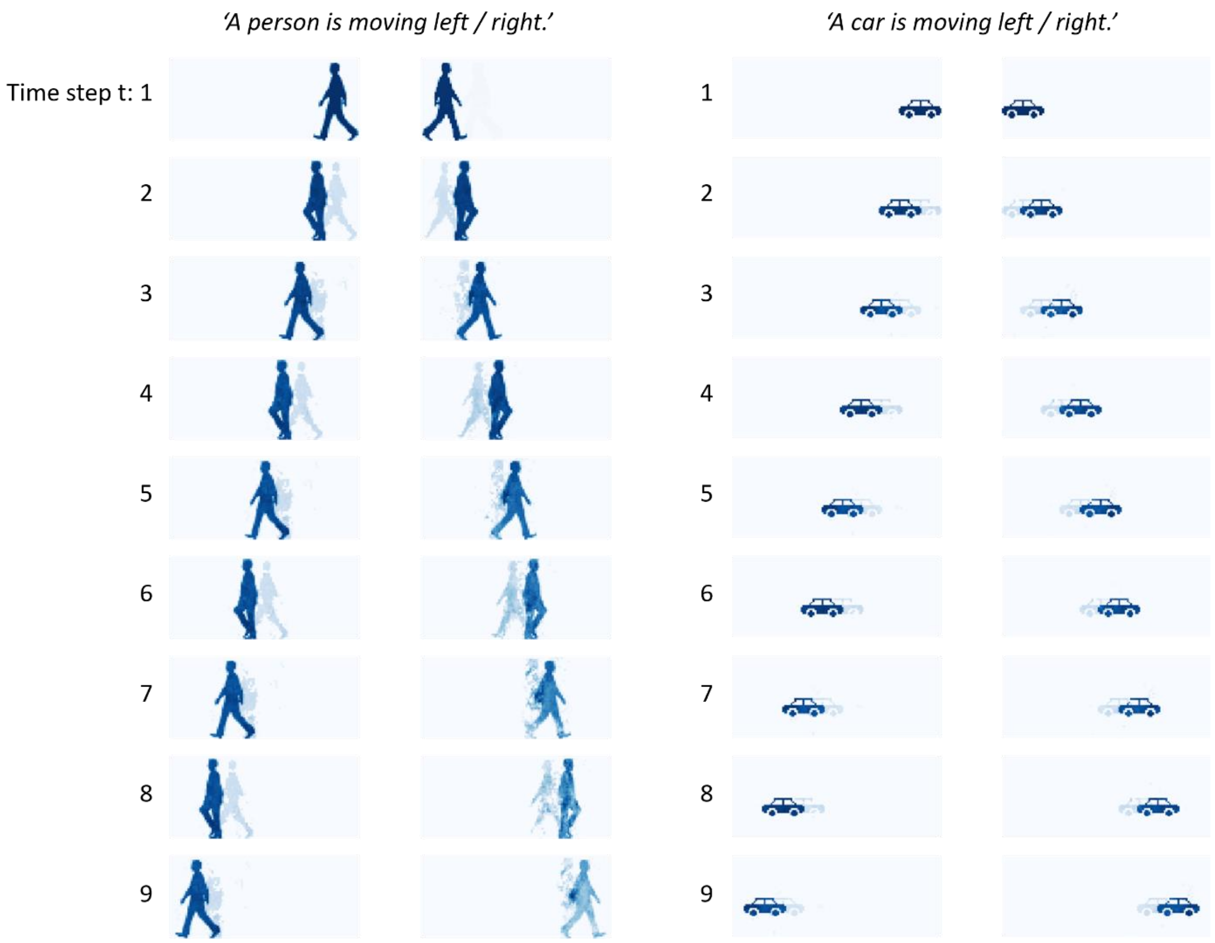

Supplementary Figure 21. Results of crossmodal audio-to-motion prediction. With audio input, a frame (X_1') of the first position is predicted (Supplementary Fig. 20). Feeding X_1' to the PMA-trained CAE results in the successful prediction of future motions. In the predicted results, the memory imprints of previous frames in *h* determines the motion direction. Predicted output images Y_t are obtained by applying a modified step function ($f(x) = 0$ (if $x < 0.4$) or x (if $x \geq 0.4$)) to *h*.

Supplementary Table 1. Comparison of our photomemristor-based system with other physical reservoir computing systems.

Structure	Mechanism	Sensing	On/off	Power/Energy per operation§	Dynamic processing	Classification	Prediction	Ref.
FeB/MgO/CoFeB	Spin-torque nano-oscillator	-	-	-	In-memory	Spoken-digit, waveform	-	3
W/WO_x/Pd	Ion migration	-	~ 10	3 nJ	In-memory	Spoken-digit	Mackey-Glass time series	4
$Ge_{15}Sb_{85}$ - based	Phase change	-	~ 10^2	< 100 nW	In-memory	Motion	-	5
Au/Cr/SnS/Cr/Au	Photogating	✓	~ 2	85 nJ	In-sensor	Language	-	6
Au/P(VDF-TrFE)/$Cs_2AgBiBr_6$/ITO	Photo-generated carriers pining	✓	-	Self-powered	In-sensor	Image, vehicle flow	-	7
ITO/ZnO/NSTO	Optoelectronic Schottky barrier	✓	~ 10^2	15 nJ#<br>30 nJ*	In-sensor or In-photomemristor	Language, Informative frame MRP		This work

§The energy per operation was calculated by V × I × t, where V is program (electrical) or reading (optical) voltage, I is the current under V, t is the pulse width.

#15 nJ was calculated from the experimental EPSC signals of motion recognition and prediction in Supplementary Fig. 13, where V = 1 V, I ≈ 300 nA, t = 50 ms.

*30 nJ was calculated from the experimental EPSC signals of words classification, in Supplementary Fig. 7, where V = 1 V, I ≈ 300 nA, t = 100 ms.

Supplementary Table 2. Comparison of our photomemristor-based system with traditional vision sensor for dynamic vision processing.

Sensor	Pixel type	Speed	Compression method	Dynamic processing	Storage	Energy per operation§	Ref.
Traditional vision sensor	Photodiode	30-240 fps	Inter-frame	Separated processor	Separated memory	Sensing: 0.42 nJ + RC: 143 nJ	8-10
Dynamic photomemristor vision sensor	Photomemristor	1-5 fps# 60 fps*	In-frame or In-photomemristor	In-photomemristor		1-5 fps: 30 nJ 60 fps: 1.5 nJ	This work

#1-5 fps indicates the speed of demonstrated dynamic vision processing in this manuscript.

*60 fps indicates the theoretical speed that was tested achievable (Supplementary Fig. 3).

§ The energy per operation in photomemristor was calculated by V × I × t, where V is program (electrical) or reading (optical) voltage, I is the current under V, t is the pulse width. The energy per operation in traditional vision sensing system was calculated by adding energy per operation of sensor (0.42 nJ)[8] and FPGA-based reservoir computing (RC) (143 nJ)[9].

Supplementary References

1. Bera, A., Peng, H., Lourembam, J., Shen, Y., Sun, X. W. & Wu, T. A versatile light-switchable nanorod memory: wurtzite ZnO on perovskite $SrTiO_3$. *Adv. Funct. Mater.* **23**, 4977-4984 (2013).
2. Fabius, J. H. & Stigchel, S. V. d. Vision while the eyes move: getting the full picture. *Sci. Adv.* **7**, eabk0043 (2021).
3. Torrejon, J., Riou, M., Araujo, F. A., Tsunegi, S., Khalsa, G., Querlioz, D., Bortolotti, P., Cros, V., Yakushiji, K., Fukushima, A., Kubota, H., Yuasa, S., Stiles, M. & Grollier, J. Neuromorphic computing with nanoscale spintronic oscillators. *Nature* **547**, 428-431 (2017).
4. Moon, J., Ma, W., Shin, J. H., Cai, F., Du, C., Lee, S. H. & Lu, W. D. Temporal data classification and forecasting using a memristor-based reservoir computing system. *Nat. Electron.* **2**, 480-487 (2019).
5. Sarwat, S. G., Kersting, B., Moraitis, T., Jonnalagadda, V. P. & Sebastian, A. Phase-change memtransistive synapses for mixed-plasticity neural computations. *Nat. Nanotechnol.* **17**, 507–513 (2022).
6. Sun, L., Wang, Z., Jiang, J., Kim, Y., Joo, B., Zheng, S., Lee, S., Yu, W. J., Kong, B.-S. & Yang, H. In-sensor reservoir computing for language learning via two-dimensional memristors. *Sci. Adv.* **7**, eabg1455 (2021).
7. Lao, J., Yan, M., Tian, B., Jiang, C., Luo, C., Xie, Z., Zhu, Q., Bao, Z., Zhong, N., Tang, X., Sun, L., Wu, G., Wang, J., Peng, H., Chu, J. & Duan, C. Ultralow-power machine vision with self-powered sensor reservoir. *Adv. Sci.* **9**, 2106092 (2022).
8. https://www.dpreview.com/news/2183540037/samsung-65-14nm-stacked-sensor-design-power-efficiency-density-mobile-image-sensors

9. Aloma, M. L. et al. Digital Implementation of a single dynamical node reservoir computer. *IEEE Trans. Circ. Syst. II* **62** 10, 977–981 (2015).

10. Abomhara, M., Khalifa, O. O., Zakaria, O., Zaidan, A. A. & Rame, A. Video compression techniques: An overview. *J. Appl. Sci.* **10**, 1834-1840 (2010).
