# Supplementary material for: Dynamic machine vision with retinomorphic photomemristor-reservoir computing
Source: Nat Commun. 2023 Apr 15;14:2169. doi: 10.1038/s41467-023-37886-y (PMC10105772; doi:10.1038/s41467-023-37886-y)
Supplement: Supplementary file 3 — Description of Additional Supplementary Files [file 41467_2023_37886_MOESM3_ESM.docx]

- Supplementary Information.
- Supplementary Movie 1. Simulated motion recognition and prediction of a symmetric object moving right at medium speed.
- Supplementary Movie 2. Simulated motion recognition and prediction of a symmetric object moving left at high speed.
- Supplementary Movie 3. Simulated motion recognition and prediction of a car moving right at high speed.
- Supplementary Movie 4. Simulated motion recognition and prediction of a person moving left at medium speed.
